# Perfused 3D angiogenic sprouting in a high-throughput in vitro platform

**DOI:** 10.1007/s10456-018-9647-0

**Published:** 2018-08-31

**Authors:** V. van Duinen, D. Zhu, C. Ramakers, A. J. van Zonneveld, P. Vulto, T. Hankemeier

**Affiliations:** 1 Division of Analytical Biosciences, LACDR, Leiden University, Leiden, The Netherlands; 2 Mimetas BV, Leiden, The Netherlands; 3 The Department of Internal Medicine, division of Nephrology and the Einthoven Laboratory for Vascular and Regenerative Medicine, LUMC, Leiden, The Netherlands

**Keywords:** Microfluidics, Angiogenic sprouting, Vascular stabilization, In vitro, 3D cell culture

## Abstract

Angiogenic sprouting, the growth of new blood vessels from pre-existing vessels, is orchestrated by cues from within the cellular microenvironment, such as biochemical gradients and perfusion. However, many of these cues are missing in current in vitro models of angiogenic sprouting. We here describe an in vitro platform that integrates both perfusion and the generation of stable biomolecular gradients and demonstrate its potential to study more physiologically relevant angiogenic sprouting and microvascular stabilization. The platform consists of an array of 40 individually addressable microfluidic units that enable the culture of perfused microvessels against a three-dimensional collagen-1 matrix. Upon the introduction of a gradient of pro-angiogenic factors, the endothelial cells differentiated into tip cells that invaded the matrix. Continuous exposure resulted in continuous migration and the formation of lumen by stalk cells. A combination of vascular endothelial growth factor-165 (VEGF-165), phorbol 12-myristate 13-acetate (PMA), and sphingosine-1-phosphate (S1P) was the most optimal cocktail to trigger robust, directional angiogenesis with S1P being crucial for guidance and repetitive sprout formation. Prolonged exposure forces the angiogenic sprouts to anastomose through the collagen to the other channel. This resulted in remodeling of the angiogenic sprouts within the collagen: angiogenic sprouts that anastomosed with the other perfusion channel remained stable, while those who did not retracted and degraded. Furthermore, perfusion with 150 kDa FITC-Dextran revealed that while the angiogenic sprouts were initially leaky, once they fully crossed the collagen lane they became leak tight. This demonstrates that once anastomosis occurred, the sprouts matured and suggests that perfusion can act as an important survival and stabilization factor for the angiogenic microvessels. The robustness of this platform in combination with the possibility to include a more physiological relevant three-dimensional microenvironment makes our platform uniquely suited to study angiogenesis in vitro.

**Electronic supplementary material:**

The online version of this article (10.1007/s10456-018-9647-0) contains supplementary material, which is available to authorized users.

## Introduction

The loss of vascular integrity plays a rate-limiting role in the onset and progression of diseases such as arteriosclerosis and cancer and conditions such as chronic inflammation and ischemia [1, 2]. Therefore, detailed knowledge of the mechanisms of microvascular loss or the formation of novel vascular structures such as those generated by angiogenesis are of major importance.

Endothelial cells (ECs) respond to pro-angiogenic stimuli by differentiating into three characteristic phenotypes: tip, stalk, and phalanx cells [3–6]. Each of these phenotypes has a specific function in the development and maturation of the newly formed vasculature, and its differentiation from ECs is tightly coordinated and regulated in order to achieve functional, luminized vascular networks. After formation of a pre-mature vascular network, perfusion of the newly formed capillary initiates the final phase of angiogenesis: stabilization of the vascular network through an increase in the adherence junctions, pruning of the non-functional sprouts, and pericyte attraction to the vascular network [7–11].

In vitro models are essential to study angiogenesis in a defined and well-controlled environment. Two-dimensional in vitro models allow the study of fundamental EC biology in high-throughput, such as migration and proliferation [12]. However, since these models lack a more physiologic, three-dimensional environment, the endothelial cells fail to show many of the typical hallmarks of endothelial cells during angiogenesis in vivo [13], such as lumen formation and differentiation into tip and stalk cells. 3D cell culture models with EC growing within a matrix such as fibrin display a higher level of physiological relevance, as ECs are able to degrade the extracellular matrix, form lumen and show anastomosis between adjacent sprouts [14, 15]. Nonetheless, as such 3D cell culture models have EC mixed with an extracellular matrix, the formed lumen is not accessible or perfusable. Furthermore, possibilities to apply a stable gradient of growth factors to direct the formation of capillaries are limited.

Microfluidic devices have micrometer-sized channels that enable spatial control over cells and matrices and allow the incorporation of important biological parameters such as flow [16] and spatial–temporal gradients [17]. Microfluidics is an important emerging technique to facilitate 3D-cell culture models aimed to more faithfully mimic tissue architecture [18]. For instance, a number of microfluidic devices for microvascular modeling have been presented that allow lumen perfusion [19–30]. For an increasing number of research laboratories that study angiogenesis such microfluidic platforms are becoming their method of choice (Table 1) [31]. However, most microfluidic assays are limited in terms of scalability, standardization, and usability [32]. Many microfluidic devices need to be manufactured manually before use, which strongly limits routine adoption [33]. Furthermore, many prototypes show limited throughput per assay (*n* < 8) [18, 20, 34] and require tubings and pumps which increases complexity and limits scalability of these platforms.

**Table 1:** Comparison of in vitro assays to study angiogenesis

Type	Assay	Strengths	Weaknesses	References
2D	Scratch	Easy to perform Easy to quantify	Lacks soft substrate for the cells Migration is in 2D	[12]
	Tube formation	Cells adhere to soft substrate Self-organization into cords Reasonable throughput Tools are available for quantification	No distinct tip/stalk cell phenotype Basement membrane extracts contain significant levels of growth factors and have a high batch-to-batch variability Limited tube survival (< 2 days) High use of reagents compared to microfluidic assays Lumens not accessible nor perfusable	[13]
3D	Spheroid	Cells grow in 3D in a soft supportive matrix Endothelial cells differentiate into tip and stalk cells Clear lumen formation Fusion of sprouts is observed Laser dissection allows capture of cells Tools available to quantify the angiogenic sprouts	Lacks spatial control over gradients Higher use of reagents compared to microfluidic assays Spheroids are randomly distributed throughout gel/matrix Lumens are not accessible nor perfusable	[14, 15, 40]
	Microfluidic	Biochemical gradients can be created and maintained Lumen formation occurs early (more comparable to in vivo) Angiogenic sprouts can be perfused Spatial control over multiple cells (e.g., fibroblasts, pericytes)	Some devices require for pumps to supply flow and maintain gradients Handling and scalability issues due incompatibility with other equipment Some devices need to be manufactured by the end-user Biocompatibility of the used materials Lack of standardization Limited possibilities to extract a subset of cells	[18–30, 32, 33]

Here, we report a standardized, high-throughput cell culture platform to study angiogenesis. The platform consists of an array of 40 microfluidic devices, integrated underneath a 384-well plate. This format is compatible with standard (high content) imaging equipment. It enables the culture of individually addressable, perfusable microvessels against a patterned, three-dimensional matrix or hydrogel. To eliminate the need for pumps while increasing the robustness and scalability, passive leveling is used as a source of flow. Within this platform, reproducible gradients can be formed and maintained for multiple days. Since gradients and perfusion are two important cues during the initial sprouting and the stabilization phase in angiogenesis [3, 35], the integration of these cues in our novel platform technology makes our model uniquely suited to perform physiologically relevant studies on the formation and regression of the microvasculature in vitro.

## Methods

### Cell culture

HUVEC-VeraVec™ human endothelial cells (Angiocrine Biosciences, hVera101) were cultured in T75 flasks (Nunc™ EasyFlask, Sigma F7552) with endothelial Cell Growth Medium MV2 (Promocell, C-22022) and used at P3 till P9. Media was replaced three times a week. Cells tested negative for mycoplasma. All cell culture was performed in a humidified incubator at 37 °C and 5% CO_2_.

### Microfluidic cell culture

3-Lane microfluidic titer plates (MIMETAS OrganoPlates 4003-400B) were used for all microfluidic cell culture. Before gel seeding, every center well was filled with 50 µL hanks balanced salt solution (HBSS) to provide optical clarity and prevention of gel dehydration. Collagen type I (R&D systems, 3447-020-01) was used as 3D scaffold. A stock solution of 5 mg/mL rat tail collagen type I was neutralized with 10% 37 g/L NaHCO_3_ (Sigma, S5761) and 10% 1 M HEPES buffer (Gibco, 15630-056) to obtain a concentration of 4 mg/mL. The neutralized collagen was kept on ice until use and used within 30 min. Using a repeater pipette, 2 µL of the neutralized collagen was added into the inlet of each gel channel. To polymerize the collagen, the device was incubated for 10 min at 37 °C, 5% CO_2_. After incubation, the device was removed from the incubator and kept sterile at room temperature right before cell loading. Endothelial cells were dissociated, pelleted, and suspended in MV2 medium in a concentration of 2 × 10^7^ cells/mL. 2 µL of the cell suspension was dispensed into the perfusion inlet and incubated for 45 min at 37 °C, 5% CO_2_. After the cells attached to the bottom of the perfusion channel, 50 µL of medium was added in the perfusion inlet and outlet wells and the plates were placed on an interval rocker platform for continuous perfusion. (Perfusion rocker, MIMETAS). The rocker was set at a 7-degree inclination and 8-min cycle time. Medium was refreshed three times a week.

### Stimulation with angiogenic factors

Microvessels were first cultured for 3 days before any gradients of growth factors were applied. Growth factors were replaced every 2–3 days. Stock solutions were prepared as following: 50 µg/mL murine VEGF in MilliQ water (Preprotech, 450-32), 20 ng/mL bFGF in MilliQ water (Peprotech, 100-18B), 1 mM Sphingosine-1-Phosphate (Sigma, S9666) in 5% 1 M HCl, 95% DMSO, and 2 µg/mL PMA (Sigma, P1585) in 1% DMSO. Angiogenic factors were diluted in MV2 culture medium and used in the following concentrations: 50 ng/mL for VEGF, 50 ng/mL for bFGF, 2 ng/mL for PMA, and 500 nM for S1P.

### Sprout permeability visualization

Angiogenic sprouts were stimulated with VEGF + bFGF + PMA + S1P for 9 days. At day 4 and day 9 after stimulation, 50 µL of a 150 kDa TRITC-Dextran (Sigma 48946) solution (0.5 mg/mL in MV2 culture media) was added to the perfusion inlet well and time-lapse images were acquired at 1 min intervals using the × 10 objective.

### Immunocytofluorescent staining

During all steps of the immunofluorescent staining, the device is placed under an angle to create flow, except during staining with primary antibody. All solutions were used in quantities of 50 µL per every inlet and outlet well, unless specified otherwise. Cells were fixed using freshly prepared 3.7% formaldehyde (Sigma 252549) in PBS. 50 µL of the fixative was added to both the perfusion inlet and outlet for 15 min at room temperature (RT), followed by a wash step with 4% FBS in PBS for 5 min. After fixation, the cells were permeabilized using 0.3% Triton-X (Sigma T8787) in PBS. After washing, the microvessels were blocked for 45 min using blocking solution (2% FBS, 0.1% Tween20 (Sigma P9169), 2% BSA (Sigma A2153) in PBS). The adherence junctions were visualized using a VE-Cadherin stain (Abcam, 33168, diluted 1:1000 in blocking solution, 30 µL pipetted in the perfusion inlet, 20 µL in the perfusion outlet), which was incubated for 1 h at RT followed by 30-min incubation with Alexa Fluor 488 (ThermoFisher Scientific, A11008, 1:250 in blocking solution). To perfuse the chips with primary antibody, the device was placed on a rocker platform. After incubation with the secondary antibody, the device is washed once with washing solution, followed by nuclei staining (NucBlue Fixed cell staining, Life technologies, R37606), and the cytoskeletal marker F-actin, stained by ActinRed™ 555 ReadyProbes® (ThermoFisher Scientific, R37112) in PBS and imaged using a high content confocal microscope (Molecular Devices, ImageXpress™ Micro Confocal) at 10x magnification.

### Sprouting quantification

The average sprouting length was quantified using FIJI v. 1.52 by manual determination of the distance between the microvessel and the tip cell sprouting furthest into the gel. The sprouting length of PMA was obtained after 3 days, all other combinations after 4 days. VEGF + PMA and VEGF + S1P microvessels after 6 days of stimulation were used to quantify the median sprout number, average diameter in the minor direction, and circularity. Images were obtained from two replicates for every condition. Using a 10x objective, we acquired 180 z-steps with 1 µm spacing and obtained two adjacent sites. The orthogonal views were extracted and analyzed in the middle of the gel region. Thresholding of the vessels was automated using Weka Segmentation tool [36] (v 3.2.27). Particle analysis was performed to include particles between 10 and 10,000 µm^2^ with a circularity between 0.10 and 1.00.

## Results

### Robust gradient formation in a 3D microenvironment

**Fig. 1:**
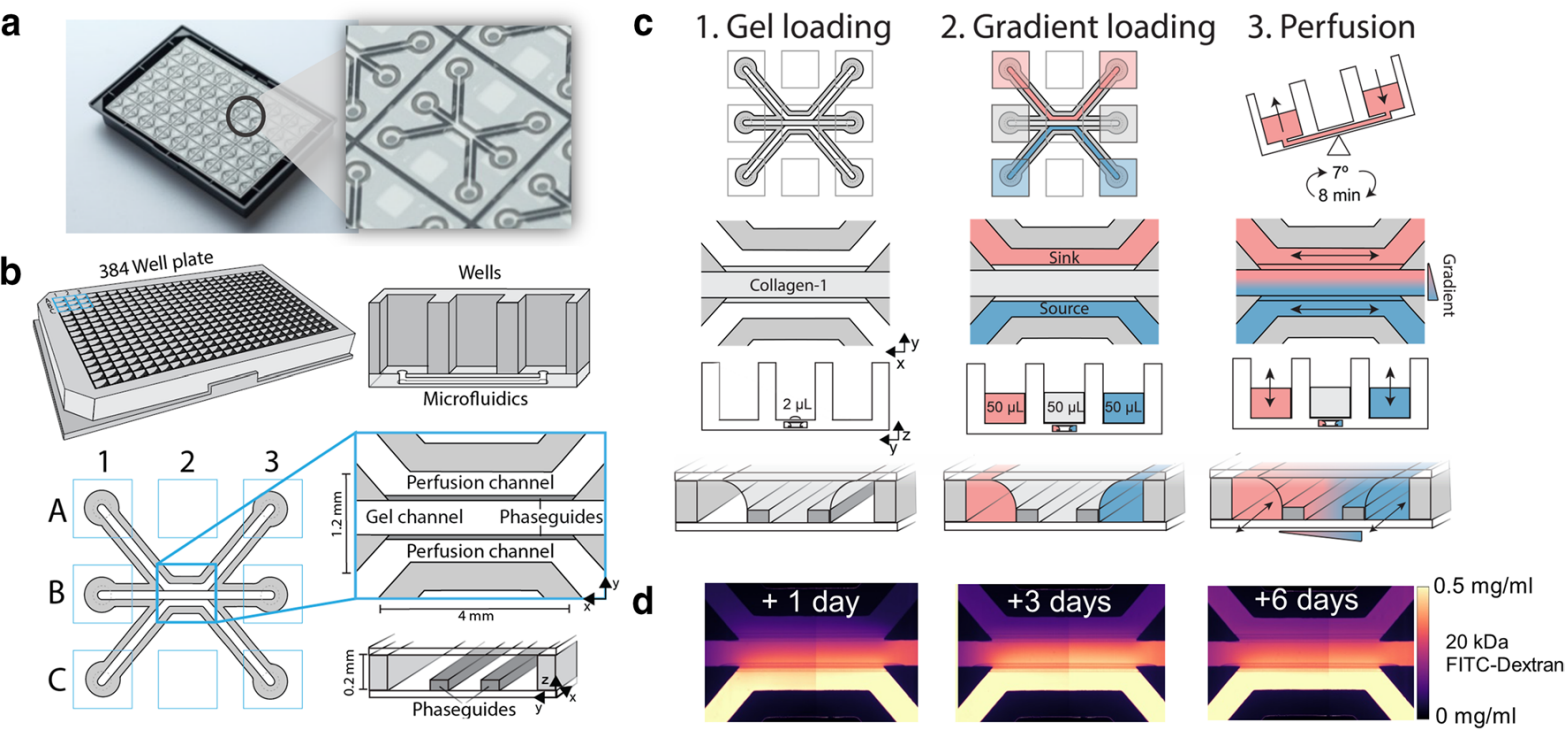
Gradient generation in a 3D microenvironment. **a** Bottom of the OrganoPlate®, a microfluidic culture platform based on a 384-well plate. The glass bottom includes 40 microfluidic devices. **b** The geometry of a single microfluidic device that is positioned underneath nine wells (3 × 3). Every device consists of three channels: one ‘gel’ channel for gel patterning, and two adjacent channels. Phaseguides prevent the patterned gel from flowing into the adjacent channels. **c** Three-step method to generate gradients in patterned hydrogels. Step 1: 2 µL of collagen-1 gel is added in the center channel and polymerized. Step 2: source and sink are added in opposite perfusion channels. Step 3: the device is placed on a rocker platform to perfuse both perfusion channels continuously to generate a gradient. **d** Gradient visualization after 1, 3, and 6 days after addition of 20 kDa FITC-Dextran as a gradient source

The microfluidic culture platform is based on a 384-well microtiter plate format. The glass bottom contains 40 microfluidic units (Fig. 1a), and each microfluidic unit is positioned underneath nine wells (3 × 3). Every unit consists of three channels: the center channel that is used to pattern an extracellular matrix (‘gel channel’) and two adjacent channels (‘perfusion channels’) (Fig. 1b). The channels are separated by PhaseGuides: small ridges that function as capillary pressure barriers, which enable patterning of cells and gel without the use of artificial membranes [37]. Every channel has one inlet and one outlet, which connect the channels with the wells in the microtiter plate. Compartmentalization is achieved by patterning a hydrogel in the middle channel (Fig. 1c, step 1), and enables the formation of gradients by adding a source and sink in the opposite perfusion channels (Fig. 1c, step 2). Without continuous replenishment of the gradients source and sink in the microfluidic channels, gradients typically last only a few minutes (data not shown). To stabilize the gradient over time, the device was placed on a rocker platform to perfuse both perfusion channels continuously and simultaneously (Fig. 1c, step 3). As the volume inside the wells is typically orders of a magnitude higher than in the microfluidic channels (the wells typically contain volumes of 50 µL, compared to < 1 µL in the microfluidic channels), the source and sink within the microfluidic channels are constant over prolonged periods of time. Thus, a stable gradient could be maintained for multiple days (Fig. 1d) without the need to replenish. Although a gradient is still present after 6 days, the steepness is affected due to saturation of the sink. Therefore, growth factors and medium were replaced at 2–3-day time intervals.

Importantly, the high hydraulic resistance of the hydrogel limits the influence of differences in hydrostatic pressures. This results in a reproducible and robust platform to generate gradients, despite the presence of small difference in volumes, for example, due to pipetting errors. Nonetheless, hydrostatic pressures still can influence the shape of the gradient, when the difference between the volumes is sufficiently large. This allows different types of gradient to be generated (e.g., linear or parabolic, Supplementary Fig. 1).

### Microvessels cultured against patterned collagen-1 gel

**Fig. 2:**
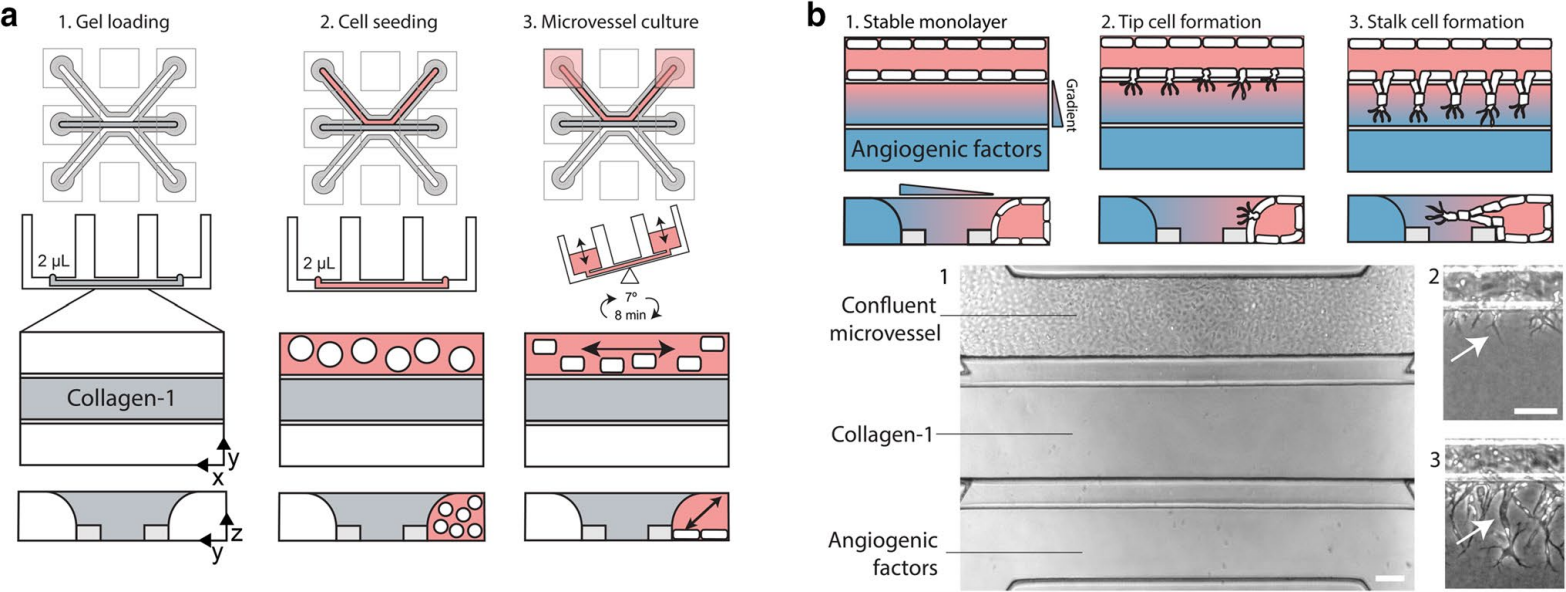
Microvessel culture against a  patterned collagen-1 gel. **a** Method the culture a microvessel within a microfluidic device. First, collagen-1 gel is patterned in the middle channel. After polymerization, an endothelial cell suspension was added in the adjacent perfusion channel. By placing the device on a rocker platform, the channels are continuously perfused. After 72 h, a confluent microvessel was formed. **b** Angiogenesis assay using a gradient of angiogenic factors. Angiogenic factors are added once a stable monolayer of ECs is formed against the gel (step 1). Addition of a gradient of angiogenic growth factors resulted in tip cells formation including filopodia at day 1 (step 2). Lumens formed by the stalk cells are visible at day 2 (step 3)

After gel loading and polymerization (Fig. 2a, step 1), endothelial cell suspensions were added to the perfusion channels adjacent to the gel. After the cells adhered to the glass substrate (step 2) of the channel, perfusion was applied by placing the device on a rocker platform (step 3). Confluent microvessels were formed after 3 days of culture, and the apical side of the vessel (the lumen) can be accessed through the perfusion channel, while the gel forms the basal side of the tube [38].

### Combination of angiogenic factors is required to induce sprouting

After reaching confluency in 3 days, the microvessels showed a stable morphology of a single monolayer against the gel (Fig. 2b, step 1), despite the numerous (angiogenic) growth factors that are present in the media (such as vascular endothelial growth factor (VEGF) and basic fibroblast growth factor (bFGF)). We included VEGF and S1P as they have been shown to induce angiogenic sprouting within a collagen-1 matrix [39–41] and included phorbol 12-myristate 13-acetate (PMA) as it has been found to promote lumen formation in the absence of fibroblasts [15, 42], and used in concentrations of 50 ng/mL for VEGF, 500 nM for S1P, and 2 ng/mL for PMA. The angiogenic growth factor cocktail was added on the basal side of the vessels, and formed a gradient within the collagen-1 gel (Fig. 2b, step 1). This induced the formation of tip and stalk cells after respectively 1 and 2 days (Fig. 2b, step 2–3).

**Fig. 3:**
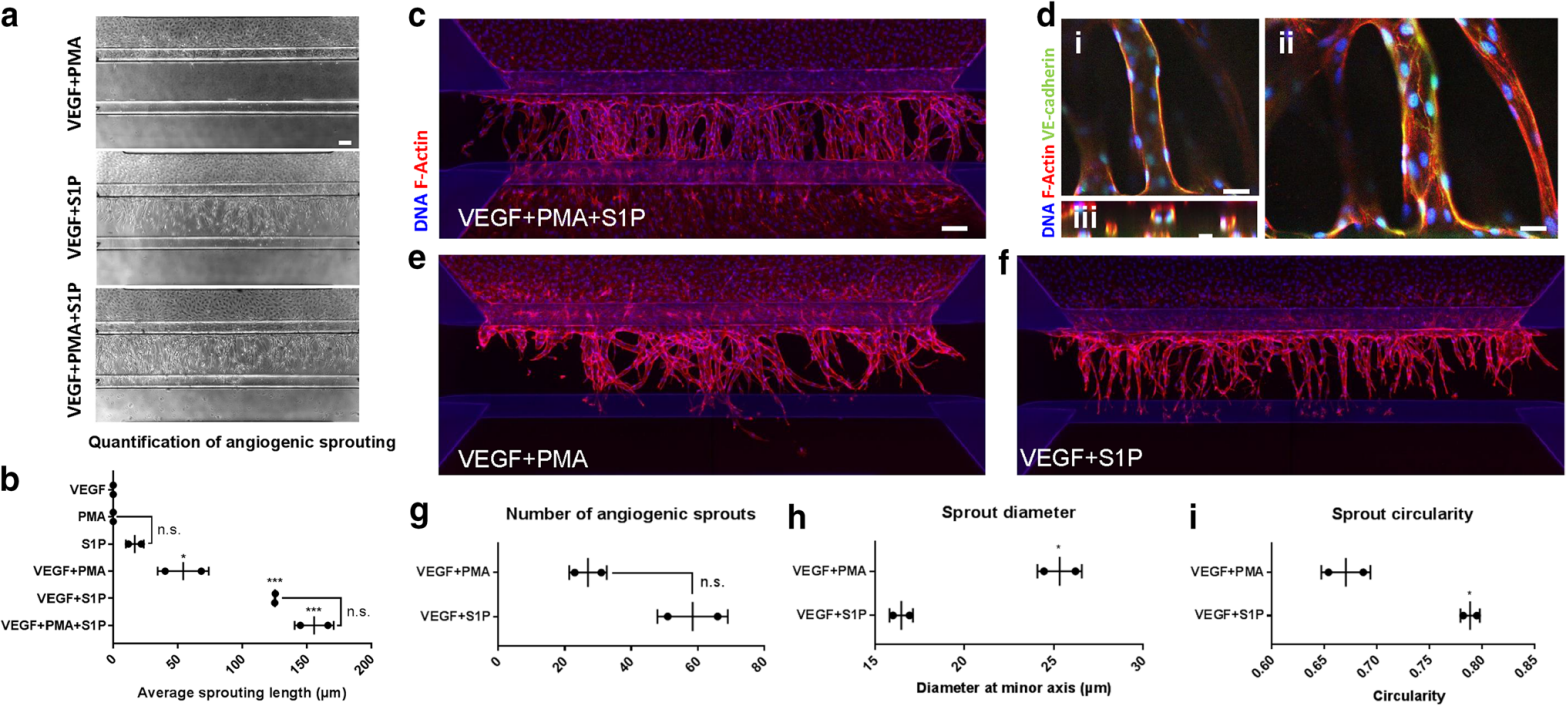
Angiogenic sprouts after addition of angiogenic factors. **a** Images of sprouting after 4 days of stimulation of a gradient of different combinations of angiogenic factors. **b** Quantification of maximum absolute sprouting length in µm after stimulation for 3 (PMA) or 4 days (all other combinations) (*n* = 6). **c** Angiogenic sprouts after 6 days of stimulation with VEGF + PMA + S1P, stained against F-actin (red) and nucleus (blue). **d** Close-up of middle (i), top (ii), and cross-section (iii) of VEGF + PMA + S1P stimulated sprouting. Stained against F-actin (red) and nucleus (blue) and VE-cadherin (green). **e** Same as **c**, but stimulation with VEGF + PMA. **f** Same as **c**, but stimulation with VEGF + S1P. **g–i** Comparison between VEGF + PMA and VEGF + S1P in number of sprouts, diameter, and circularity (*n* = 2). Significance was calculated using one-way anova (**b**) or Student’s *t* test (**g**–**i**) and shown as n.s (non-significant), *(*P* < 0.05), **(*P* < 0.01), or ***(*P* < 0.001). Scale bars: 100 µm. Graphs are presented as mean ± SD

Interestingly, adding either VEGF, S1P, or PMA alone on the basal side did not result in angiogenic sprouting (Supplementary Fig. 2). We quantified the angiogenesis after addition of various combinations of VEGF, PMA and S1P (Fig. 3a, b). VEGF + PMA + S1P together resulted in angiogenesis including tip/stalk cell formation, the presence of filopodia and lumen formation and directional growth towards the gradient. The sprouts fully traversed the gel after about 6 days and started to form a continuous monolayer against in the channel on the other side of the gel and in the basal perfusion channel  (Fig. 3c). The angiogenic sprouts have a clear lumen formation (Fig. 3d, panel i), appear circular in a cross-sectional view (Fig. 3d, panel ii), and have clear VE-cadherin expression (Fig. 3d, panel iii).

To identify the contribution of PMA and S1P to angiogenic sprouting, we directly compared VEGF + PMA with VEGF + S1P. The combination of VEGF + PMA triggered the formation of angiogenic sprouts into the gel, but the tip cells fail to develop their characteristic tip cell morphology including filopodia and the sprouts lack directionality after 6 days of sprouting (Fig. 3e and Supplementary Fig. 3a, b). Furthermore, the sprouts appear to be non-homogenously distributed within the collagen gel. In contrast, VEGF + S1P shows sprouts that are also connected the sprouts to the main vessel, but sprouts are equally distributed within the gel with a clear directionality towards the gradient (Fig. 3f). Although there were not significantly more sprouts after VEGF + S1P stimulation (Fig. 3g), the diameter of the sprouts was significantly lower (Fig. 3h). We quantified the circularity of the sprouts to estimate the directionality: a perpendicular sprout appears circular in a cross-sectional view with a value closer to 1, while a deviating sprout appears flattened (closer to 0). This shows that VEGF + S1P sprouts have a significantly higher circularity and thus improved directionality towards the gradient compared to VEGF + PMA (Fig. 3i). Taken together, these results clearly demonstrate that in a gradient-driven, 3D cell culture environment, a combination of different cues is required to trigger angiogenesis, and S1P is a crucial factor in the distribution and guidance during angiogenic sprouting.

### Anastomosis triggers remodeling and stabilization

**Fig. 4:**
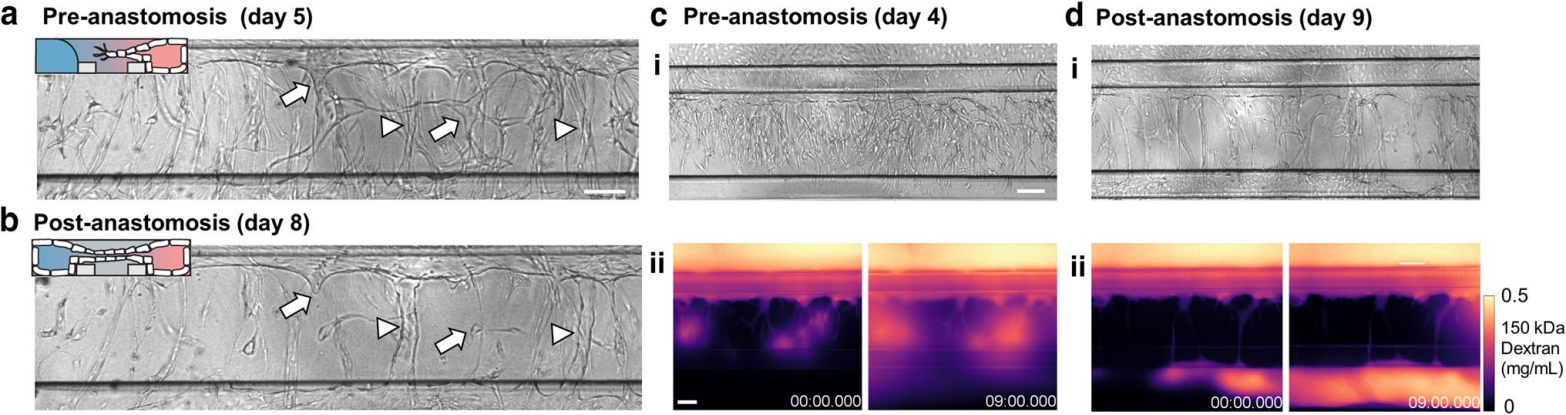
Anastomosis with basal channel triggers pruning and maturation of angiogenic sprouts. **a** Angiogenic sprouts 5 days after addition of VEGF + PMA + S1P. Compared to the angiogenic sprouts at day 8. **b** Some sprouts regressed (arrows) while other sprouts remain and showed increased lumen diameter (arrowheads). **c** Angiogenic sprouts after 4 days of stimulation invaded into the gel but are not yet connected to the bottom perfusion channel. Fluorescent images were obtained every minute and directly after addition of a 0.5 mg/mL 150 kDa TRITC-Dextran solution in culture media. Panel ii shows the pseudo-colored fluorescent images after 0 and 9 min after addition of the dextran solutions. Time is indicated in min. **d** Same as in **c**, but after 9 days of stimulation. Sprouts are connected to the other side and formed a confluent microvessel in the basal perfusion channel. Scale bars: 100 µm

Prolonged exposure to growth factors caused the angiogenic sprouts to anastomose, and connection is formed between the two perfusion channels. After anastomosis, we observed a significant reduction of sprouts (Fig. 4a, b). Some angiogenic sprouts display the characteristic steps involved in pruning: first, the lumen collapses, followed by regression of the angiogenic sprouts towards the parental vessel (Fig. 4a, b, arrows), while other angiogenic sprouts remained and increased their lumen diameter (Fig. 4a, b arrowheads).

The formation of perfusable lumen within the sprouts is visualized by perfusion of the main vessel with 0.5 mg/mL 150 kDa TRITC-Dextran (Fig. 4c, d). A surplus of 50 µL is added to the inlet well, which fills the parental vessels and flows into the angiogenic sprouts. When angiogenic sprouts did not connect to the basal perfusion channel (Fig. 4c), spots were visible within the collagen where dextran leaks out of the tip of the sprouts (panel ii, left, 0 min). These spots increased over time (right, 9 min). However, after anastomosis (Fig. 4d), sprouts retained the dextran in their lumen, and shows subsequent filling of the bottom basal perfusion channel. This shows that sprouts stabilize and form a functional barrier after a connection has been formed.

## Discussion

We report a robust, standardized microfluidic cell culture platform to study gradient-driven angiogenesis of a perfused microvessel in high-throughput. Each device contains 40 individually addressable microfluidic units and enables the culture of 40 identical microvessels. An important advantage of this assay is the defined geometry of the microfluidic channels, as this results in reproducible experimental cell culture conditions (position and density of the cells, amount of flow, position of the extracellular matrix and the shape of the gradient) and increases the robustness and scalability of our assay.

Perfusion in our device is induced by passive leveling using a rocker platform, and has two crucial advantages. First, the flow is simultaneously applied throughout all microfluidic units, which results in reproducible gradient formation. Second, as tubing and pumps are not required the throughput is greatly increased: the assay is scalable since multiple experiments can be performed by stacking of culture platforms on top of each other. Nonetheless, using a rocker platform to induce flow is also a trade-off that has its downsides: first, the requirement of a rocker platform limits us to perform time-lapse imaging only at discrete time points, as the vessels and gradient require continuous perfusion. Second, vasculature in vivo is exposed to continuous, unidirectional flow that is an important mechano-biological signal in during angiogenesis [43], while flow in this assay occurs at discrete time points and is bi-directional. Thus, despite the evidence that flow affects the remodeling and maturation of the capillaries in our model, the exact contribution of flow in this assay is difficult to determine.

We showed that gradient-driven angiogenic sprouting through an extracellular matrix requires not just the presence of VEGF, but a combination of multiple angiogenic factors [44]. The combination of VEGF + PMA + S1P was the most optimal cocktail to trigger quick, robust, directional angiogenesis with angiogenic sprouts with clear lumen formation. VEGF + PMA showed a random distribution of the sprouts and an absence of filopodia on the tips cells, and the sprouts lacked directionality. In contrast, a VEGF + S1P gradient showed formation of angiogenic sprouts, including tip cells with filopodia. Filopodia allow the tip cells to sense a biochemical gradient [4], and explains the observed directionality of the angiogenic sprouts. This suggests that S1P plays an important role in the differentiation into functional tip cells and the observed repetitive formation of angiogenic sprouts. Such a repetitive formation of angiogenic sprouts can be explained by a reaction–diffusion mechanism between VEGF and Flt-1, the soluble form of VEGF receptor. Stalk cells are known to secrete Flt-1, which binds VEGF and prevents neighboring cells to become tip cells [45]. This is required for efficient angiogenic sprouting into the matrix [3], with evenly distributed sprouts roughly every 100 µm, as predicted in silico [8, 9]. It has been shown that S1P has a pro-angiogenic effect in vitro [39, 40, 46–48] and in vivo [39, 49, 50]. Our data suggest a pro-angiogenic synergy between S1P and VEGF, which is in agreement with the fact that inhibition of S1P also prevents VEGF-induced angiogenesis in vivo [51]. Interestingly, S1P is also known for its barrier stabilizing, anti-angiogenic properties, and vascular maturation [52, 53]. Therefore, we hypothesize that the effect of S1P is dependent on whether it is present on the apical side of ECs (lumen) or basal side, either mediated by differences in apical and basal expression of S1P receptors [54] or by dimerization with other receptors, like basally expressed VEGFR2 [46]. A better understanding of the precise mechanisms of S1P signaling in angiogenesis will provide therapeutic strategies that specifically target the pro-angiogenic effects of S1P [49].

Prolonged exposure (> 6 days) to a gradient of angiogenic stimuli resulted in sprouts that connect the two perfusion channels (anastomosis). This connection resolves the gradient, as there is a direct connection between the source and sink, and also results in the onset of flow through the sprouts. There remains controversy about the exact mechanism that leads to pruning. In vivo, this is either shear-mediated or due to changing receptor expression after a resolved (oxygen) gradient [10, 55]. Once anastomosis occurred, we observed remodeling of the capillary bed, including pruning and regression of angiogenic sprouts within the collagen. Furthermore, some sprouts increased in lumen diameter, likely caused by the onset of perfusion [56]. By controlling shear levels and oxygen tension in this assay, we will be able to determine which of those effects is the crucial mechanism in pruning.

Perfusion of the sprouts with fluorescently labeled dextran showed that angiogenic sprouts that did anastomose are permeable near the tip/stalk cell region. In contrast, anastomosed sprouts retained the 150 kDa dextran solution within their lumen, suggesting that the connection between the two channels triggers maturation of the ECs in the sprouts, as they adopt their characteristic phalanx phenotype including mature cell–cell junctions [55, 57]. Furthermore, once the angiogenic sprouts connected, the medium can be switched back to the original culture medium with low levels or growth factors, while the integrity of the sprouts remained (Supplementary movies 2, 3), which suggests that perfusion is an important survival factor for angiogenic sprouts in the absence of a high concentration of angiogenic factors like VEGF.

We expect that our platform will be widely adopted for a range of applications, including both fundamental studies of the mechanisms of angiogenesis as well as for the identification of factors involved in microvascular destabilization or regression such as observed in for example diabetic retinopathy, nephropathy, macular degeneration, heart failure, and tumor angiogenesis. The platform can be used to assess disease parameters on a high-throughput scale and can be expanded to comprise other cell types such as stromal cells of the tissue or organ of interest.

## Conclusion

We demonstrate a gradient-driven, three-dimensional angiogenesis assay in a standardized microfluidic platform. Angiogenic sprouting is induced from a perfused microvessel through a patterned collagen-1 gel. The combination of angiogenic factors was optimized to trigger angiogenic sprouting that faithfully reproduces all the angiogenic events that occur in vivo, such as the differentiation of the endothelial cells into tip, stalk, and phalanx cells and the formation of perfusable lumen. It was found that a combination of VEGF, S1P, and PMA provided the optimal cocktail for 3D angiogenic sprouting. After the angiogenic sprouts anastomosed through the collagen to the other channel, remodeling and stabilization of the capillary bed was observed.

## Electronic supplementary material

Below is the link to the electronic supplementary material.

**Supplementary Figure 1: Shaping a gradient using hydrostatic pressure. a)** Using double the volumes in the gel inlet and gel outlet compared to the perfusion inlets and outlets resulted in a parabolic gradient shape. **b)** In contrast, equal volumes in all the wells result in a linear gradient shape. The gradient is visualized 4 hours after addition of 20 kDa FITC-Dextran. Fluorescence intensity is measured at the center of the gel, over the complete width of the gel and plotted accordingly. (TIF 3373 KB)

**Supplementary figure 2: Angiogenic sprout growth over time after stimulation with various angiogenic factors**. Microvessels were grown for 3 days and stimulated for 4 days using different angiogenic factors. (TIF 11251 KB)

**Supplementary figure 3: Sprout morphology over time under culture different conditions. a)** Phase contrast images of sprouts of after 4,5 and 6 days of stimulation **b)** Fixed microvessels after 6 days of stimulation with VEGF+PMA or VEGF+S1P and stained against F-actin (red) and nuclei (blue). (TIF 13604 KB)

**Supplementary figure 4: Method used to quantify the sprout number, diameter and circularity from multiple z-slices**. (TIF 1127 KB)

**Supplementary Figure 5: Influenced of gradient shape by the permeability of the cell barrier. a)** Gradient profile 24 hr after permeabilization with VEGF+PMA+S1P. The dotted line indicates the position of the cell monolayer. **b)** The gradient is restored and comparable to a gradient without cells. **c)** A region of interest is defined to quantify the gradient within the gel. **d)** The change in intensity in the y-direction (arrow) is used to calculate the slope of the gradient. **e)** Comparison of the slope of the gradient between different conditions (n=2) shows that the gradient in a system with more permeable cell layers is not significantly different compared to gel control (P=0.13). Bars represent mean±sd. (TIF 1234 KB)

Supplementary material 6 (AVI 1193 KB)

Supplementary material 7 (MP4 1296 KB)

Supplementary material 8 (MP4 1305 KB)
