# Supplementary figures and images for: Perfused 3D angiogenic sprouting in a high-throughput in vitro platform
Source: Angiogenesis. 2018 Aug 31;22(1):157–65. doi: 10.1007/s10456-018-9647-0 (PMC6510881; doi:10.1007/s10456-018-9647-0)

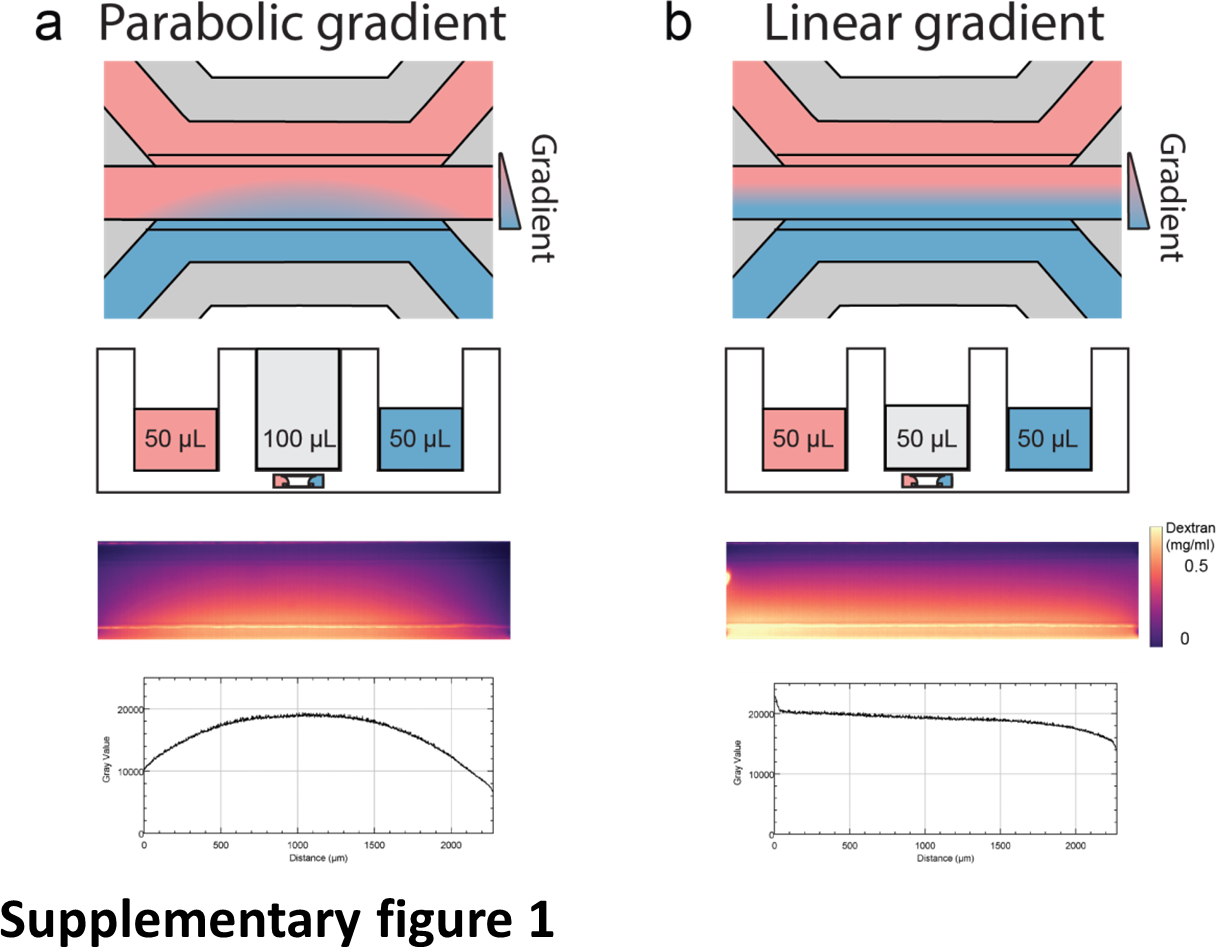

Supplement: Supplementary file 1 — Supplementary Figure 1: Shaping a gradient using hydrostatic pressure. a) Using double the volumes in the gel inlet and gel outlet compared to the perfusion inlets and outlets resulted in a parabolic gradient shape. b) In contrast, equal volumes in all the wells result in a linear gradient shape. The gradient is visualized 4 hours after addition of 20 kDa FITC-Dextran. Fluorescence intensity is measured at the center of the gel, over the complete width of the gel and plotted accordingly. (TIF 3373 KB) [file 10456_2018_9647_MOESM1_ESM.tif]

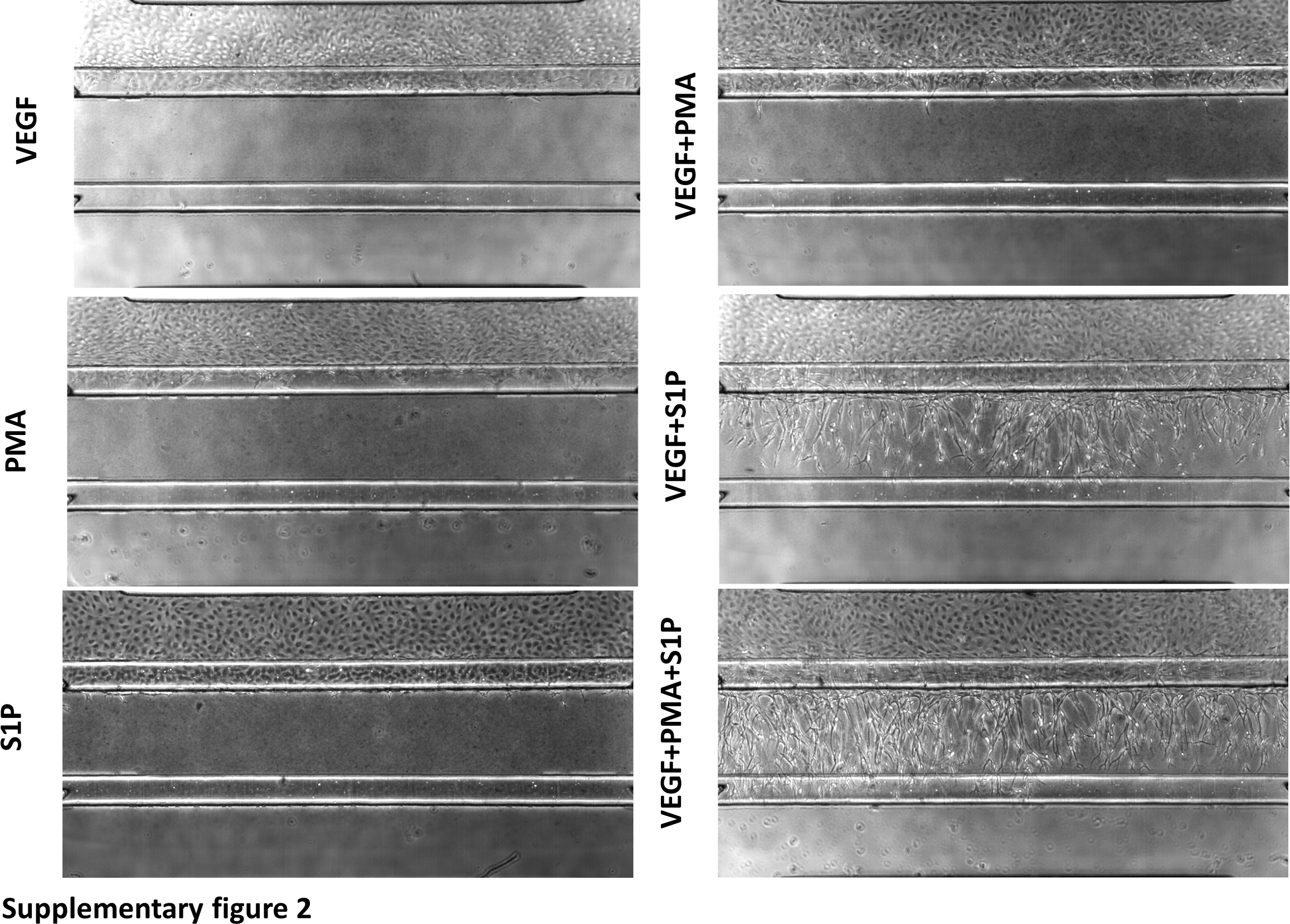

Supplement: Supplementary file 2 — Supplementary figure 2: Angiogenic sprout growth over time after stimulation with various angiogenic factors. Microvessels were grown for 3 days and stimulated for 4 days using different angiogenic factors. (TIF 11251 KB) [file 10456_2018_9647_MOESM2_ESM.tif]

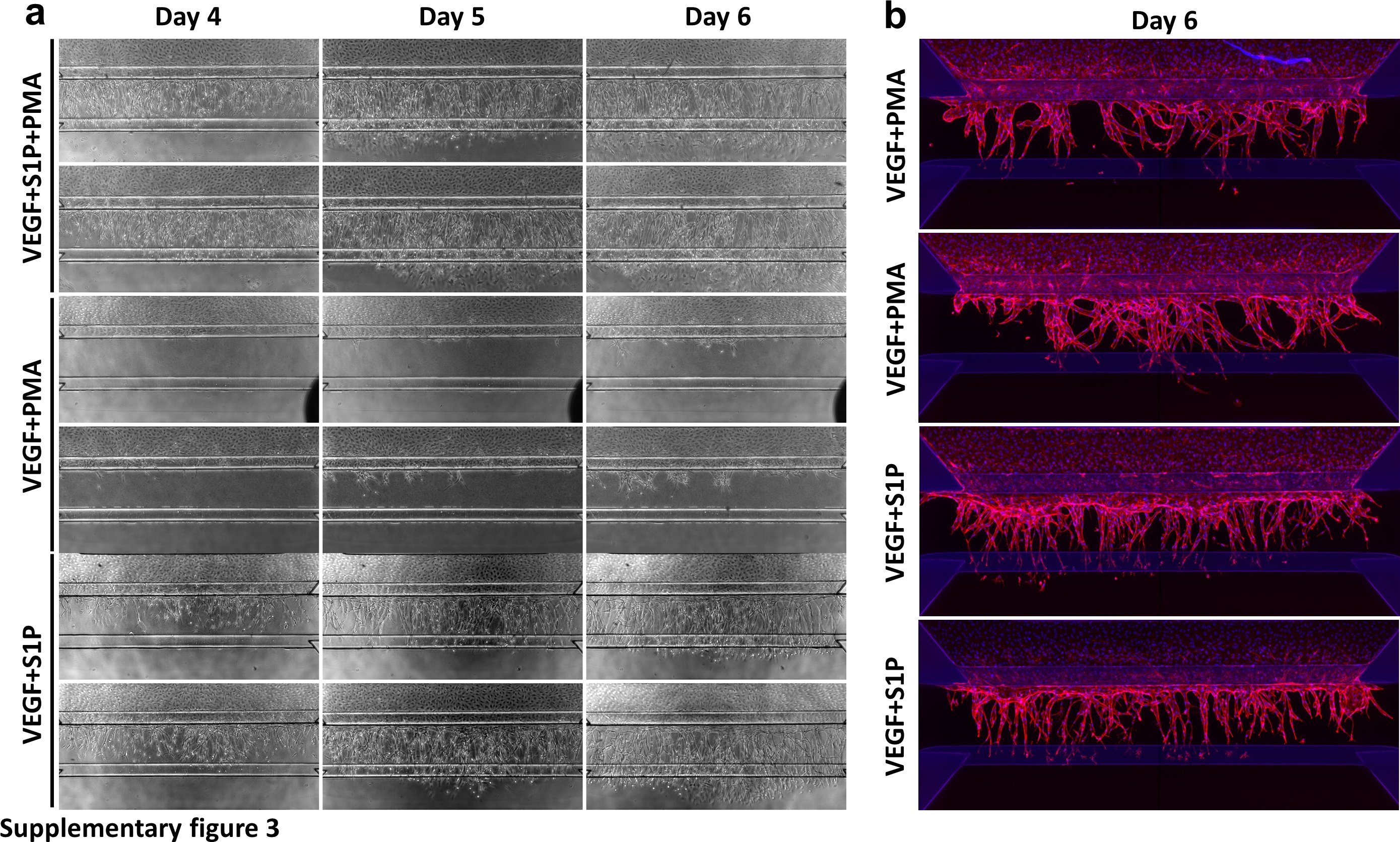

Supplement: Supplementary file 3 — Supplementary figure 3: Sprout morphology over time under culture different conditions. a) Phase contrast images of sprouts of after 4,5 and 6 days of stimulation b) Fixed microvessels after 6 days of stimulation with VEGF+PMA or VEGF+S1P and stained against F-actin (red) and nuclei (blue). (TIF 13604 KB) [file 10456_2018_9647_MOESM3_ESM.tif]

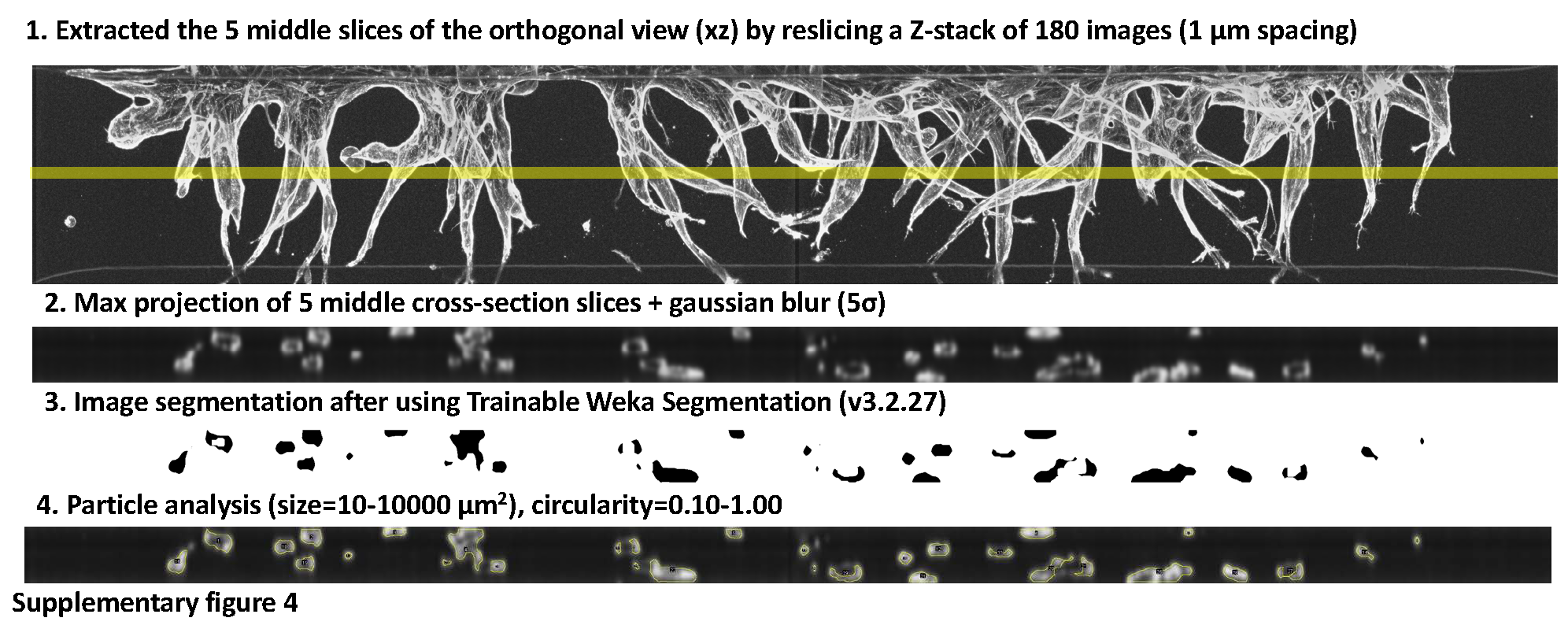

Supplement: Supplementary file 4 — Supplementary figure 4: Method used to quantify the sprout number, diameter and circularity from multiple z-slices. (TIF 1127 KB) [file 10456_2018_9647_MOESM4_ESM.tif]

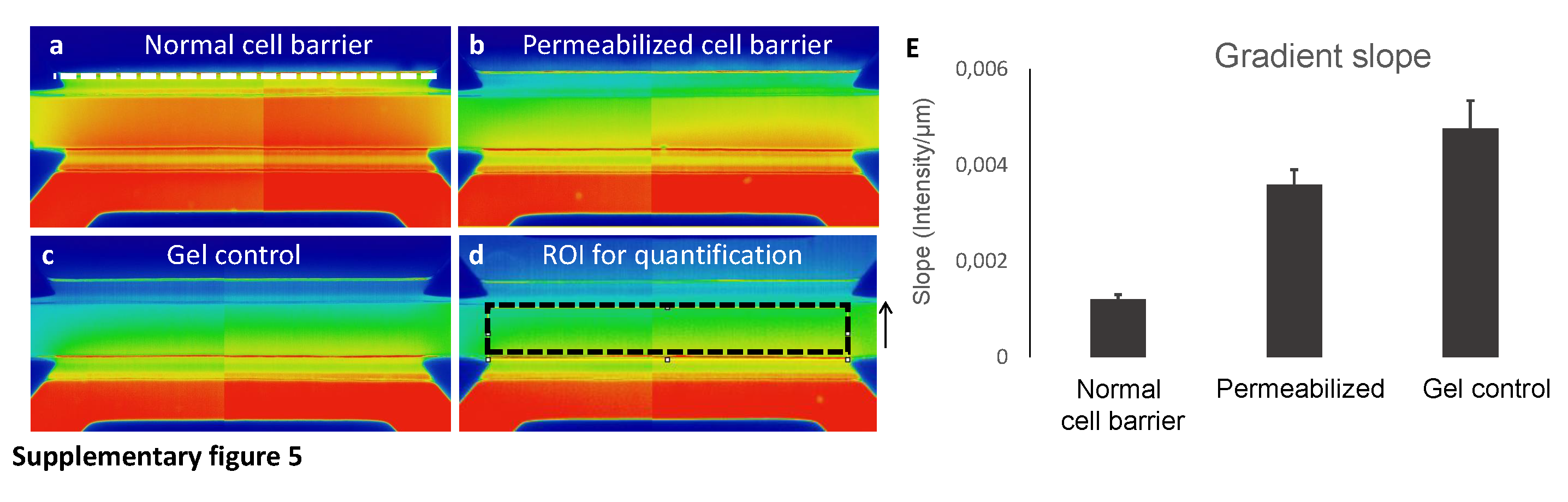

Supplement: Supplementary file 5 — Supplementary Figure 5: Influenced of gradient shape by the permeability of the cell barrier. a) Gradient profile 24 hr after permeabilization with VEGF+PMA+S1P. The dotted line indicates the position of the cell monolayer. b) The gradient is restored and comparable to a gradient without cells. c) A region of interest is defined to quantify the gradient within the gel. d) The change in intensity in the y-direction (arrow) is used to calculate the slope of the gradient. e) Comparison of the slope of the gradient between different conditions (n=2) shows that the gradient in a system with more permeable cell layers is not significantly different compared to gel control (P=0.13). Bars represent mean±sd. (TIF 1234 KB) [file 10456_2018_9647_MOESM5_ESM.tif]
